# A double-edged sword: interactions of CX_3_CL1/CX_3_CR1 and gut microbiota in systemic lupus erythematosus

**DOI:** 10.3389/fimmu.2023.1330500

**Published:** 2024-01-17

**Authors:** Rana A. Estaleen, Christopher M. Reilly, Xin M. Luo

**Affiliations:** ^1^ Department of Biomedical Sciences and Pathobiology, Virgnia-Maryland College of Veterinary Medicine, Virginia Tech, Blacksburg, VA, United States; ^2^ Biomedical Sciences, Edward Via College of Osteopathic Medicine, Blacksburg, VA, United States

**Keywords:** systemic lupus erythematosus, lupus, lupus nephritis, CX3CR1, gut microbiota, autoimmune disease, autoimmunity

## Abstract

Systemic lupus erythematosus (SLE) is a systemic chronic disease initiated by an abnormal immune response to self and can affect multiple organs. SLE is characterized by the production of autoantibodies and the deposition of immune complexes. In regard to the clinical observations assessed by rheumatologists, several chemokines and cytokines also contribute to disease progression. One such chemokine and adhesion molecule is CX_3_CL1 (otherwise known as fractalkine). CX_3_CL1 is involved in cell trafficking and inflammation through recognition by its receptor, CX_3_CR1. The CX_3_CL1 protein consists of a chemokine domain and a mucin-like stalk that allows it to function both as a chemoattractant and as an adhesion molecule. In inflammation and specifically lupus, the literature displays contradictory evidence for the functions of CX_3_CL1/CX_3_CR1 interactions. In addition, the gut microbiota has been shown to play an important role in the pathogenesis of SLE. This review highlights current studies that illustrate the interactions of the gut microbiota and CX_3_CR1 in SLE.

## 1 Introduction

**Figure 1:**
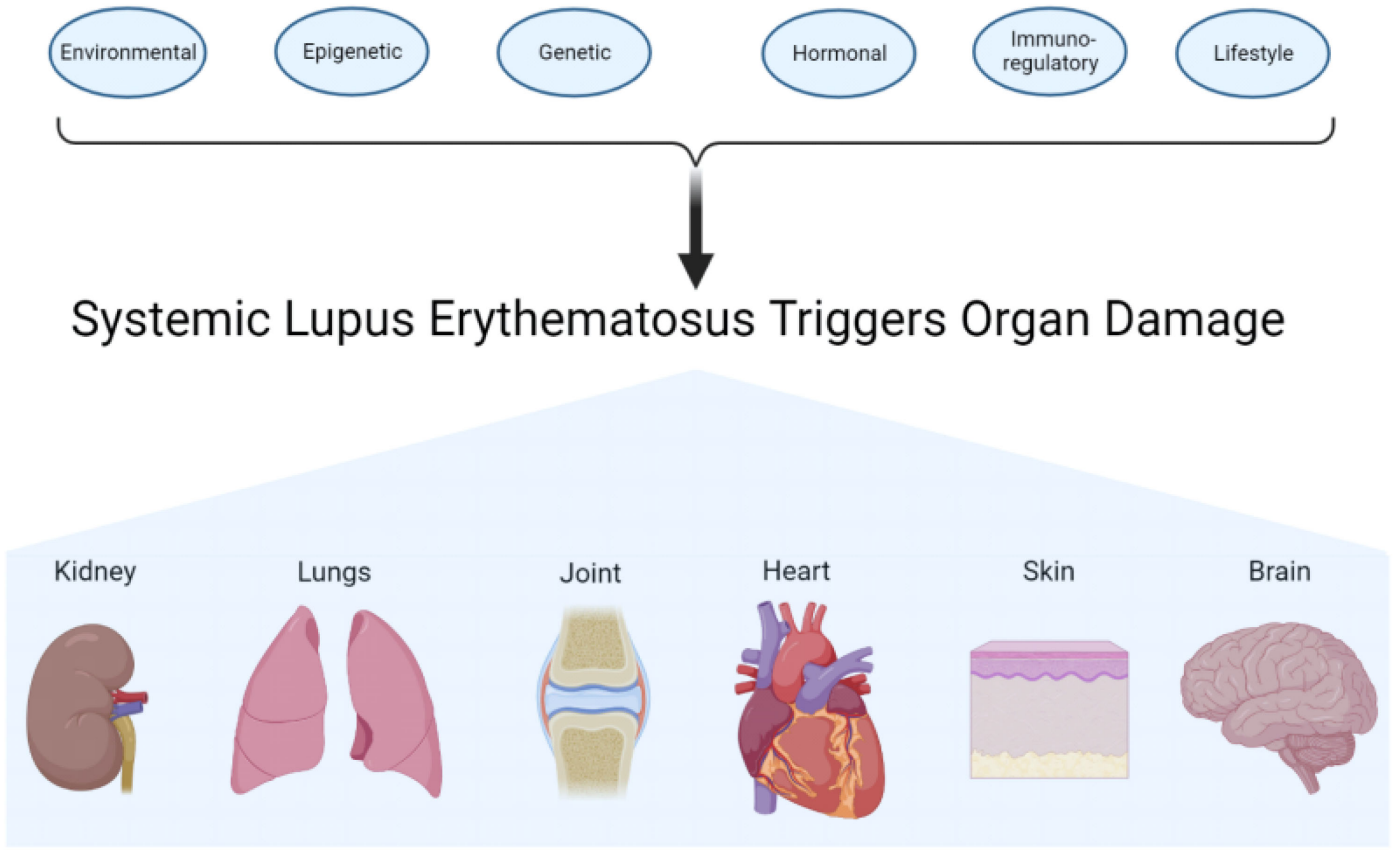
Contributing factors and target organs of systemic lupus erythematosus. Environmental, epigenetic, genetic, hormonal, immunoregulatory, and lifestyle factors work on the immune system. The actions of the different factors affect and may also damage different organs or tissues.

Systemic lupus erythematosus (SLE), also known as lupus, is a chronic autoimmune disease that can affect multiple organs in the body (1, 2). The disease is caused by an abnormal autoimmune response (1, 3). Normally, the body’s immune system works to protect against foreign invaders. In SLE, the immune system becomes hyperactive, producing antibodies that attack normal tissues and organs, some of which include the skin, blood, heart, lungs, joints, kidneys, and brain (2, 3). SLE is diagnosed when a patient meets 4 out of 11 diagnostic criteria, established by the American College of Rheumatology and the European League Against Rheumatism. It is one of the most heterogeneous diseases treated by physicians, presenting challenges to the diagnosis as well as establishment of proper treatments (4). Lupus is characterized by “on” and “off” periods known as flares and remission, respectively. These are when the patient endures periods of illness and periods of wellness, respectively (3, 5). Phenotypic expression of lupus varies between individuals from different ethnicities (6), with incidence rates ranging between 20 and 200 cases per 100,000 persons. There is a higher prevalence in individuals of African, Asian, and Hispanic backgrounds or ancestries (2, 6). SLE affects both men and women; however, the disease is much more frequent among women than men (6), particularly in women of childbearing age who are diagnosed nine times more frequently than men (2). In more economically developed countries, the 5-year survival rate is over 95% in both adults and children; however, in less economically developed countries, the survival is significantly lower in both populations (7). Although the cause of SLE is unknown (3), there are some factors that influence the development and/or severity of SLE, such as lifestyle, environmental, genetic, epigenetic, hormonal, and immunoregulatory factors (**Figure 1**).

## 2 Roles of CX_3_CL1/CX_3_CR1 in SLE

Chemokines are a group of molecules that recruit leukocyte subsets under homeostatic and pathological conditions (8). The chemokine ligand superfamily is divided into two subgroups: the CC chemokine family, which includes 28 members, and the CXC chemokine family, which includes 16 members. They interact with chemokine receptors expressed on the cell surface. Chemokine receptors are G-protein-coupled receptors and can promote target cells to adhere to the endothelium or direct their movement to their destination based on the concentration gradient of a given chemokine (9). Chemokine C-X_3_-C motif ligand 1, CX_3_CL1, also known as fractalkine, binds to its seven transmembrane G-protein-coupled receptor CX_3_CR1 (10). CX_3_CR1 was discovered, using fractalkine–alkaline phosphatase fusion protein in 1997, as a receptor with a high affinity for CX_3_CL1 that is expressed by and lymphocytes and monocytes (10).

CX_3_CL1 is produced by the renal tubular epithelium in humans (11), and it can be found on leukocytes, blood monocytes, phagocytes, and T cells in both humans and mice (12). CX_3_CL1 has a soluble form and a transmembrane form, which function to induce chemotaxis and adhesion of CX_3_CR1^+^ leukocytes, respectively (8). CX_3_CL1/CX_3_CR1 interaction has an antiapoptotic effect that sustains the survival of CX_3_CR1^+^ leukocytes (8, 13). CX_3_CR1 is found on several types of leukocytes. It has high expression on CD16^+^ natural killer cells, and its expression is upregulated by IL-2 in human CD4^+^ and CD8^+^ T cells (10).

In healthy individuals, CX_3_CR1 is required for atherogenesis and homeostasis of monocytes by promoting cell survival (13). It was reported that in the absence of the chemokine receptor or its ligand, fractalkine, there was a significant reduction in Gr1^low^ blood monocytes levels under both steady-state and inflammatory conditions. This suggests that the interaction between CX_3_CL1 and CX_3_CR1 is an essential survival signal given that their absence would lead to monocyte death (13). Of note, Ly6G and Ly6C, previously referred to as Gr1, are markers of myeloid differentiation. Neutrophils express Ly6G and Ly6C; in addition, dendritic cells and subpopulations of lymphocytes and monocytes express Ly6C (14, 15). Gr1^+^ cells, under steady-state conditions, can be found in the bloodstream, contributing to immune surveillance (16). In inflammatory conditions, Gr1 cells have increased mobilization of Ly6C^+^ monocytes from bone marrow to bloodstream (16). This contributes to monocyte recruitment and migration to the kidney, which leads to kidney injury (17). In this aspect, the reduction of Gr1^low^ blood monocytes in the absence of CX_3_CL1 and CX_3_CR1 interaction may be beneficial, although it is unclear if the decrease of monocytes in blood is partly due to increased recruitment to tissues. The latter, obviously, suggests a protective role of CX_3_CL1 and CX_3_CR1 interaction in blocking the mobilization of Gr1^low^ monocytes from the blood to the kidney. Therefore, in the context of lupus, the absence of CX_3_CL1/CX_3_CR1 interaction may facilitate migration of Gr1^+^ inflammatory monocytes to the kidney, causing injury.

However, studies reported that an antagonist of CX_3_CL1 delayed the onset and slowed the progression of lupus nephritis in MRL/*lpr* mice (18), suggesting a detrimental role for CX_3_CL1/CX_3_CR1 interaction in lupus. On the other hand, we demonstrated that the treatment of *Lactobacillus* spp. attenuated splenomegaly and renal lymphadenopathy via a CX_3_CR1-dependent mechanism (19), suggesting that CX_3_CR1 may be used as a target for therapeutics. Moreover, CX_3_CR1 may locally prevent profibrotic macrophage retention, thus reducing kidney fibrosis (20). Additionally, CX_3_CL1 acts as a chemoattractant and adhesion molecule in glomerulonephritis (21). **Table 1** illustrates a dichotomy in the literature concerning CX_3_CL1/CX_3_CR1 activation. These studies taken together show that the CX_3_CL1/CX_3_CR1 interaction can be considered to be a “double-edged sword” due to its involvement of both the pathogenesis and protection of renal diseases. Notably, some studies were performed in lupus-like mouse models and further research is needed for human lupus nephritis.

**Table 1:** Roles of CX_3_CL1/CX_3_CR1 in the kidney as pathogenesis promoters or regulators.

Promotion of Pathogenesis	Regulation of Pathogenesis
CX_3_CR1 facilitates macrophage movement to the kidney and supports ischemic acute renal failure in mice (22).	Through a CX_3_CR1-dependent mechanism, *Lactobacillus* spp. treatment attenuated splenomegaly and renal lymphadenopathy in murine lupus (19).
CX_3_CL1 expression recruits CX_3_CR1^+^ cells and prolong glomerulonephritis (21).	The presence of CX_3_CR1 potentially reduces kidney fibrosis (20).
CX_3_CR1 antagonism delays the onset and ameliorates the progression of lupus nephritis in mice (18).	The CX_3_CR1-dependent adhesion mechanism protects against kidney damage during sepsis, via Ly6C^high^ monocytes (23).
Systemic activation of CX_3_CL1/CX_3_CR1 contributes to chronic kidney disease-associated cardiovascular disease (24).	Loss of function of CX_3_CR1 leads to exacerbation of chronic kidney disease compared to patients that do not have this loss of function (25).
The aortic CX_3_CL1/CX_3_CR1 axis is upregulated in chronic kidney disease in mice (26).	*Cx3cr1* deficiency exacerbated glomerulonephritis in MRL/*lpr* mice (27).
Kidney damage by hypertension leads to upregulation of CX_3_CL1 and CX_3_CR1 (28).	
*CX3CR1* polymorphism may be involved in the pathogenesis of end-stage renal disease (29).	

## 3 Roles of gut microbiota in SLE

**Figure 2:**
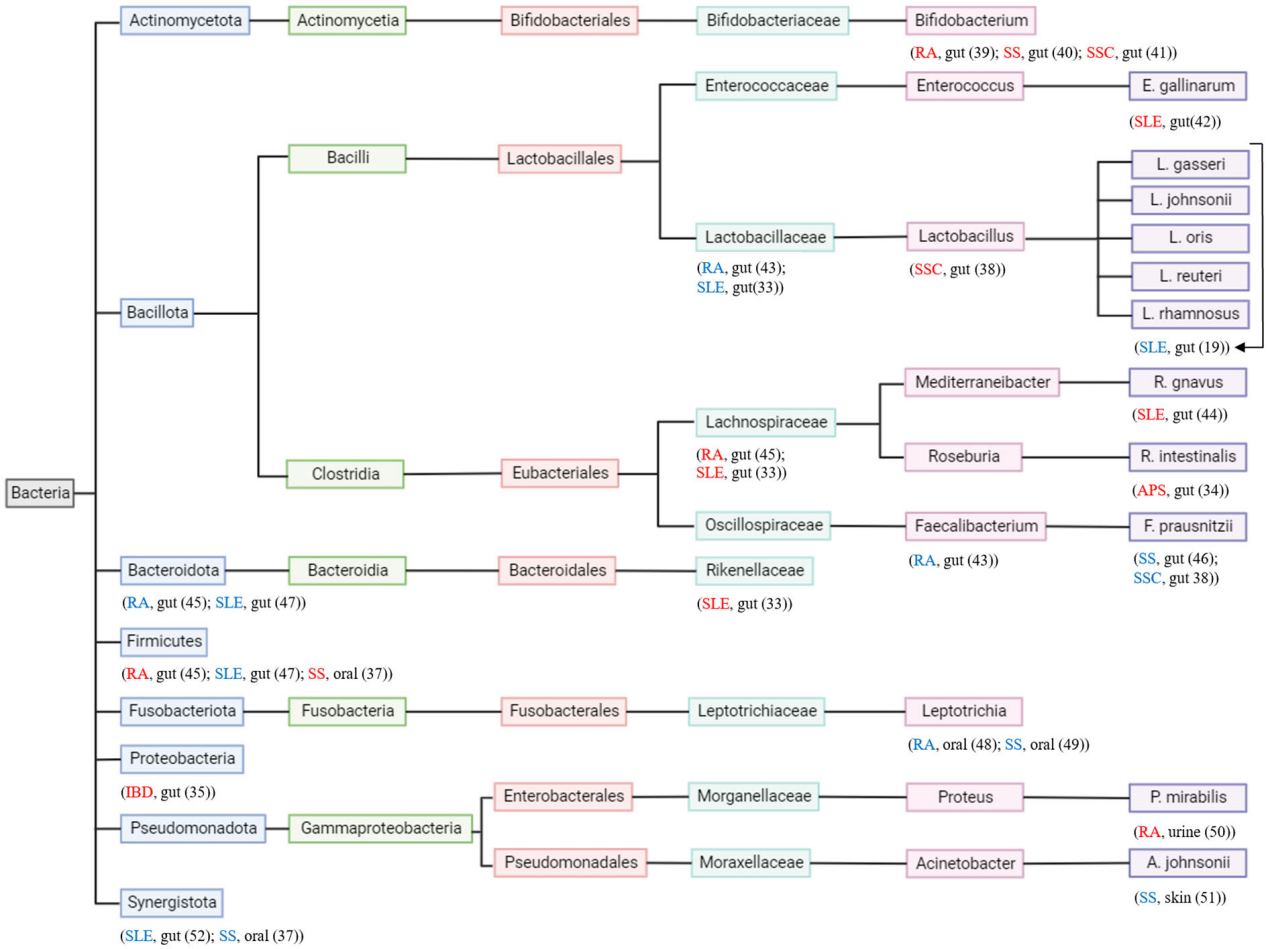
Phylogenetic tree of bacteria and autoimmune diseases. Each taxonomy level has a different color. Autoimmune disease and where in the body the bacteria is found are located under the studied bacterium. Autoimmune disease in “red” indicates the bacteria contributes to its pathogenesis and autoimmune disease in “blue” indicates the bacteria contributes to its attenuation.

It has been recognized that a healthy gut microbiome contributes to the health of the host (30). Microbiota is the microorganisms’ entire population that colonizes a specific space, which includes archaea, bacteria, fungi, protozoans, and viruses (31). These microbes occupy different organs or systems such as the gut, mouth, skin, and vagina (32). The microbiota engages in many features of normal host physiology, from nutritional status to stress and behavioral responses. Moreover, they can contribute to many diseases and affect different organs (31). The immune system and bacteria have a meticulous relationship that maintains a balance to control inflammation (19). Gut microbiota dysbiosis has been reported in numerous autoimmune diseases (33), including antiphospholipid syndrome (APS) (34), inflammatory bowel disease (IBD) (35), rheumatoid arthritis (RA) (36), SLE (33), Sjogren’s syndrome (SS) (37), and systemic sclerosis (SSC) (38). **Figure 2** illustrates different bacteria altered in autoimmune diseases.

A study of lupus nephritis linked disease activity to a gut commensal. Fecal microbiota of SLE patients was tested for pathobionts using 16S ribosomal RNA (rRNA) analyses, and it was found that SLE patients possessed a microbiome that had a decrease in species richness diversity as well as a reduction in taxonomic complexity (44). *Ruminococcus (Blautia) gnavus* was found to be significantly higher in SLE patients than in healthy individuals (44). *R. gnavus* was further investigated in a longitudinal analyses of lupus gut microbiota to study microbiota resilience and disease activity. *R. gnavus* was found to be expanded during high disease activity, and it was detected in almost half of patients during lupus nephritis disease flares. After whole-genome sequence analysis of *R. gnavus*, it was found that there were 34 genes that help adapt and expand within a host with inflammation (53). This rationalizes that these bacteria that are found expanded during disease flares possess the necessary tools to withstand inflammation.

Toll-like receptor (TLR) 7.1 transgenic (Tg) C57BL/6 mice were cohoused with wild-type littermates (WTLs). This revealed an enrichment of *Lactobacillus*, *Desulfovibrio*, and Rikenellaceae in TLR7.1 Tg mice. When WTL mice were colonized with microbiota containing these pathobionts from TLR7.1 Tg mice, they showed an increase in leaky gut with translocation of pathobionts (54). Thus, the enrichment of these bacterial communities exacerbates SLE pathogenesis. Moreover, fecal transfer from dysbiotic gut microbiota of triple congenic (TC) lupus-prone mice into germ-free congenic C57BL/6 mice induced autoimmune phenotypes when TC donor mice exhibited autoimmunity (55). Again, this shows the significance of how the gut microbiota affects SLE pathogenesis.

The role of gut microbiota in renal pathogenesis of SLE has not been widely investigated. We found that the gut microbiota of MRL/*lpr* lupus-prone mice has significant depletion of Lactobacillales as disease develops. A weekly treatment of a mixture of five *Lactobacillus* strains (*Lactobacillus gasseri*, *L. johnsonii*, *L. oris*, *L. reuteri*, and *L. rhamnosus*) attenuated lupus-like clinical signs, including splenomegaly and lymphadenopathy, and prolonged survival (19). Individually, the different strains did not have an effect, but the mixture of *Lactobacillus* spp. acted synergistically to attenuate lupus-like disease. Mechanistically, the mixture of *Lactobacillus* spp. increased the percentages of the effector memory T cells in the spleen and mesenteric lymph nodes while decreasing the percentages of the central memory T cells and double-negative T cells. The mentioned outcomes suggest that in order to attenuate lymphadenopathy and splenomegaly, *Lactobacillus* spp. may act on T cells (19). Further investigations will determine if the above results can be replicated in human SLE patients. Furthermore, *Lactobacillus* spp. may increase FOXP3-negative Tr1 cells in the spleen and mesenteric lymph nodes to control inflammation (19). The treatment of *Lactobacillus* reversed a “leaky” gut, which the MRL/*lpr* mice possessed. The treatment also promoted an anti-inflammatory environment by decreasing IL-6 and increasing IL-10 production in the gut. In the circulation, IL-10 was increased whereas IgG_2a_ was decreased (the latter of which is believed to be a major immune deposit in the MRL/*lpr* kidney). Furthermore, T cells showed a Treg-Th17 balance toward the Treg phenotype in the kidney (2).

In another study, fecal microbiota transplantation from untreated active SLE female patients vs. healthy female patients to germ-free (GF) mice was investigated (56). SLE patients’ fecal microbiota caused GF mice to develop a series of lupus-like phenotypic features, including imbalanced cytokines, upregulation of SLE-related genes, autoimmune antibodies, and altered distribution of immune cells in mucosal and peripheral immune response. Importantly, these results depict a causal role of aberrant gut microbiota in influencing the pathogenesis of SLE (56). A 12-week investigation of the safety and efficacy of fecal microbiota transplantation to treat SLE patients was also explored (57). SLE patients were treated with oral encapsulated fecal microbiome from healthy donors. Upon completion of this study, it was concluded that fecal microbiota transplantation is safe and effective for SLE patients. Also, they found that fecal microbiota transplantation alters the gut microbiome and modifies the short-chain fatty acid metabolic profile in SLE patients (57).

There was a decrease in the Firmicutes/Bacteroidetes ratio in SLE patients (47). This balance between the two phyla in the human gut microbiota is dependent on the individual’s physiology. This ratio is important because dysbiosis of these two phyla in the intestines is associated with SLE (47). Another study has shown that the frequency of Synergistetes positivity correlated with the Firmicutes/Bacteroidetes ratio in healthy individuals but reduced in SLE patients’ fecal samples with an increase in anti-double stranded DNA (dsDNA) titers (52). This may suggest a protective role that the intestinal bacterium Synergistetes has on humoral immunity. Notably, many studies, including ours, have used 16S rRNA sequencing that can only be accurate at the genus level. Genus-level microbiota evaluation is likely insufficient to ideally link mechanisms of pathobionts to SLE disease. Metagenomic shotgun sequencing, which can reach species and even the strain level, will be more useful.

## 4 Interaction of CX_3_CR1 and gut microbiota in SLE

CX_3_CR1^+^ cells are present in tissues lining the intestine, and phagocytes expressing CX_3_CR1 can clear pathogenic bacteria from the gut lumen (58). When the chemokine is absent, the integrity of the intestinal barrier is compromised (12). This phenomenon results in an altered microbiome as well as endotoxemia and aggravation of the gut and liver inflammation (59, 60). This suggests that in SLE, CX_3_CR1 may play a protective role in clearing pathogenic gut bacteria and, in its absence, the gut lining may be compromised, which will cause a leaky gut and bacteria to interact with peripheral organs, consequently causing inflammation.

Using the MRL/*lpr* lupus nephritis model, we observed the enhancement of gut mucosal barrier with Lactobacillales treatment that resulted in less bacteria translocation across the intestinal epithelium (2). This led to reduced activation and migration of CX_3_CR1^+^ antigen-presenting cells to lymph nodes. Furthermore, the Lactobacillales treatment significantly reduced *Cx3cr1* in the mesenteric lymph node, which would suggest that the treatment may reduce the migration of antigen-presenting cells to the mesenteric lymph node (2).

We have demonstrated that *Cx3cr1*-deficient mice have a noticeably different gut microbiota from *Cx3cr1* ^+/+^ mice (27). The gut microbiota of *Cx3cr1*-deficient mice was corrected with *Lactobacillus* administration, and consequently, glomerulonephritis was reversed (27). This suggests that CX_3_CR1 plays an important role in glomerulonephritis in MRL/*lpr* mice through a gut microbiota-dependent mechanism. We demonstrated that the treatment of *Lactobacillus* spp. attenuated splenomegaly and renal lymphadenopathy via a CX_3_CR1-dependent mechanism (19), suggesting that CX_3_CR1 may be used as a target for therapeutics.

In C57BL/6 mice, the depletion of the gut microbiota via broad-spectrum antibiotics, which was reversible by fecal transplantation, decreased the levels of CX_3_CR1 in macrophages and bone marrow monocytes (61). This suggests that the gut microbiota plays a role in the induction of CX_3_CR1 in addition to activation of downstream pathways.

**Figure 3:**
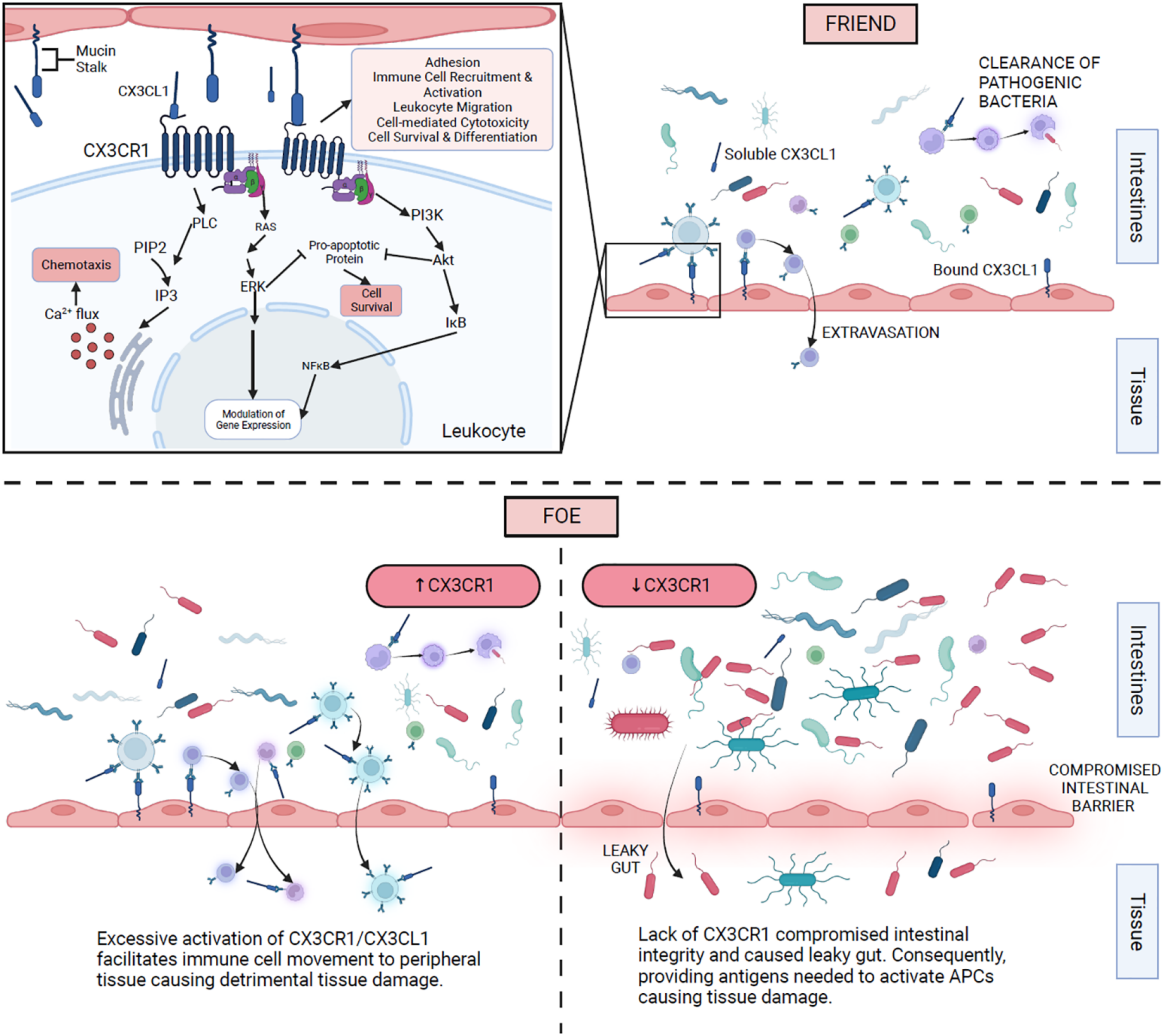
Proposed mechanisms of action of CX_3_CR1 and gut microbiota in SLE. The top right section shows CX_3_CR1 interaction with membrane-bound and soluble CX_3_CL1 on different immune cells. The top left section illustrates the intracellular mechanism of action once CX_3_CR1 is activated. The bottom right section expresses the consequences of the absence of CX_3_CR1. The bottom left section displays the effects of excessive activation of CX_3_CR1.

**Figure 3** illustrates different mechanisms of action for CX_3_CR1 as it interacts with CX_3_CL1 and the gut microbiota in SLE.

CX_3_CR1 has not been studied in context with the gut microbiota in human SLE. We speculate that a single loss-of-function nucleotide polymorphism of *CX3CR1* in humans may result in an exacerbation of lupus nephritis. Further investigation of CX_3_CR1 is needed in order to replicate the mentioned findings in human SLE patients.

## 5 Conclusion and future directions

While the cause of SLE remains unknown, there are factors that can influence its severity, including environmental, epigenetic, genetic, hormonal, immunoregulatory, and lifestyle factors. When SLE is in the “on” phase of the disease, it may affect the kidney, lungs, joints, heart, skin, and/or brain. We and others have established that the gut microbiota is a causative factor in SLE instead of the result. Indeed, immune cells help maintain the gut barrier and protect against pathogens entering the body. The CX_3_CR1 receptor helps maintain the integrity of the gut barrier (12). The data are more in support of its protective properties in SLE. Finally, the gut microbiota and CX_3_CR1 interact to influence one another in various pathways.

The pathways by which the gut microbiota and CX_3_CR1 interact are not well studied. Investigations suggest that the gut microbial dysbiosis causing a leaky gut activates CX_3_CR1-expressing cells, which subsequently increase inflammation, thus causing aggregated spleen and/or kidney inflammation. It was reported that a single loss-of-function nucleotide polymorphism of *CX_3_CR1* leads to exacerbation of chronic kidney disease compared to patients that do not have this loss of function (25). An altered gut microbiome may lead to lupus nephritis, and when combined with the loss of function of CX_3_CR1, it may increase the risk of renal failure.

The contradictions in **Table 1** are pronounced studies that ought to be investigated further for a better understanding of CX_3_CL1/CX_3_CR1 interactions, particularly on how they can be used therapeutically. A potential treatment of the leaky gut in lupus is to agonize CX_3_CR1 in antigen-presenting cells, which may activate these cells to clear any bacteria leaking from the gut. Future investigations may shed light on whether CX_3_CR1 is a friend or foe in SLE. This may be addressed by blocking CX_3_CR1 or its ligand, CX_3_CL1, and assess disease progression. Another way to investigate the role of CX_3_CR1 is to have a conditional knockout in an SLE model and dissect the mechanism of action of CX_3_CR1 in a cell- or tissue-specific manner.

## Author contributions

RE: Writing – original draft. CR: Writing – review & editing. XL: Writing – review & editing.
